# Stochastic Cellular Fate Decision Making by Multiple Infecting Lambda Phage

**DOI:** 10.1371/journal.pone.0103636

**Published:** 2014-08-08

**Authors:** Matthew L. Robb, Vahid Shahrezaei

**Affiliations:** Department of Mathematics, Imperial College, London, United Kingdom; Tata Institute of Fundamental Research, India

## Abstract

Bacteriophage lambda is a classic system for the study of cellular decision making. Both experiments and mathematical models have demonstrated the importance of viral concentration in the lysis-lysogeny decision outcome in lambda phage. However, a recent experimental study using single cell and single phage resolution reported that cells with the same viral concentrations but different numbers of infecting phage (multiplicity of infection) can have markedly different rates of lysogeny. Thus the decision depends on not only viral concentration, but also directly on the number of infecting phage. Here, we attempt to provide a mechanistic explanation of these results using a simple stochastic model of the lambda phage genetic network. Several potential factors including intrinsic gene expression noise, spatial dynamics and cell-cycle effects are investigated. We find that interplay between the level of intrinsic noise and viral protein decision threshold is a major factor that produces dependence on multiplicity of infection. However, simulations suggest spatial segregation of phage particles does not play a significant role. Cellular image processing is used to re-analyse the original time-lapse movies from the recent study and it is found that higher numbers of infecting phage reduce the cell elongation rate. This could also contribute to the observed phenomena as cellular growth rate can affect transcription rates. Our model further predicts that rate of lysogeny is dependent on bacterial growth rate, which can be experimentally tested. Our study provides new insight on the mechanisms of individual phage decision making. More generally, our results are relevant for the understanding of gene-dosage compensation in cellular systems.

## Introduction

Bacteriophage Lambda is a temperate virus that infects the bacteria *Escherichia coli*. Upon infection, the cell undergoes one of two fates. In the lytic fate, the phage replicate quickly and kill the cell, whereas in the lysogenic fate the phage become dormant, replicating slowly along with the bacterial replication. The lysogenic state is very stable [1]–[3] but under specific induction conditions the phage can re-enter the lytic pathway. It is thought that the lysis-lysogeny decision is a response to prevent extinction by lying dormant in malnourished cells or when there is an overabundance of phage [4]. The genetic circuit controlling this decision making process is long studied and serves as a paradigm for the study of genetic regulation and biological switches [5]–[8]. Although we do not yet have a complete understanding of lambda phage developmental decision making and stability, mathematical modelling has been quite influential in producing mechanistic insight [1], [7]–[15].

Biochemical reaction networks are stochastic due to low copy number of participating biomolecules and the fluctuations in the cellular environment [16]. The stochastic dynamics will inevitably influence the decision making processes in cellular systems [17], [18]. One of the earliest mathematical studies of stochastic dynamics in genetic networks focused on the probabilistic nature of the lambda phage switch and showed that clonal cells in similar environments can still exhibit different fates due to gene expression noise [11]. Game theoretic arguments suggest probabilistic cell fate determination may minimise the chance of phage extinction [19].

The probabilistic choice between lysis and lysogeny can be affected by multiple factors. Classic experiments by Kourilsky showed that physiological state of the cell, such as starvation can significantly affect the probability of lysogeny [20]. In particular the number of phage simultaneously infecting the bacteria, the so called multiplicity of infection (MOI), increases the probability of lysogeny [20]–[22]. It has also been observed that an increase in cell volume (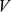) at the time of infection results in significant decrease in the probability of lysogeny [23]. Analysis of simple deterministic models of the lysis-lysogeny genetic circuit shows that in fact the outcome of the decision should depend on the concentration of viral genes, which is related to MOI divided by 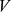 [12], unifying the experimental observations mentioned above. However, a more recent study used single phage resolution and time-lapse imaging to follow the fate of individual bacteria as it is infected at different MOI and showed that the decision depends not only on the viral concentration (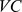) but also directly on MOI [24]. This observation can be interpreted as evidence for independent decision making by individual phage [24]. An alternative interpretation of the observed results is the existence of partial gene dose compensation in the phage-bacteria system [14]. However, neither of these interpretations provide a mechanistic explanation of direct dependence of the outcome of decision on MOI.

In this study, we set out to investigate possible biophysical mechanisms that underlie the specific dependence of probability of lysogeny on MOI observed recently [24]. We use stochastic simulation of a simple model of the lambda phage genetic switch [12] to study the lysis-lysogeny decision process. We reveal an interplay between the decision making threshold and molecular noise that can provide an explanation for the results. We also investigate possible contributions of spatial and cell cycle effects. We conclude by discussing other possible contributing factors, experimentally testable predictions and the broader relevance of our results.

## Results

**Figure 1:**
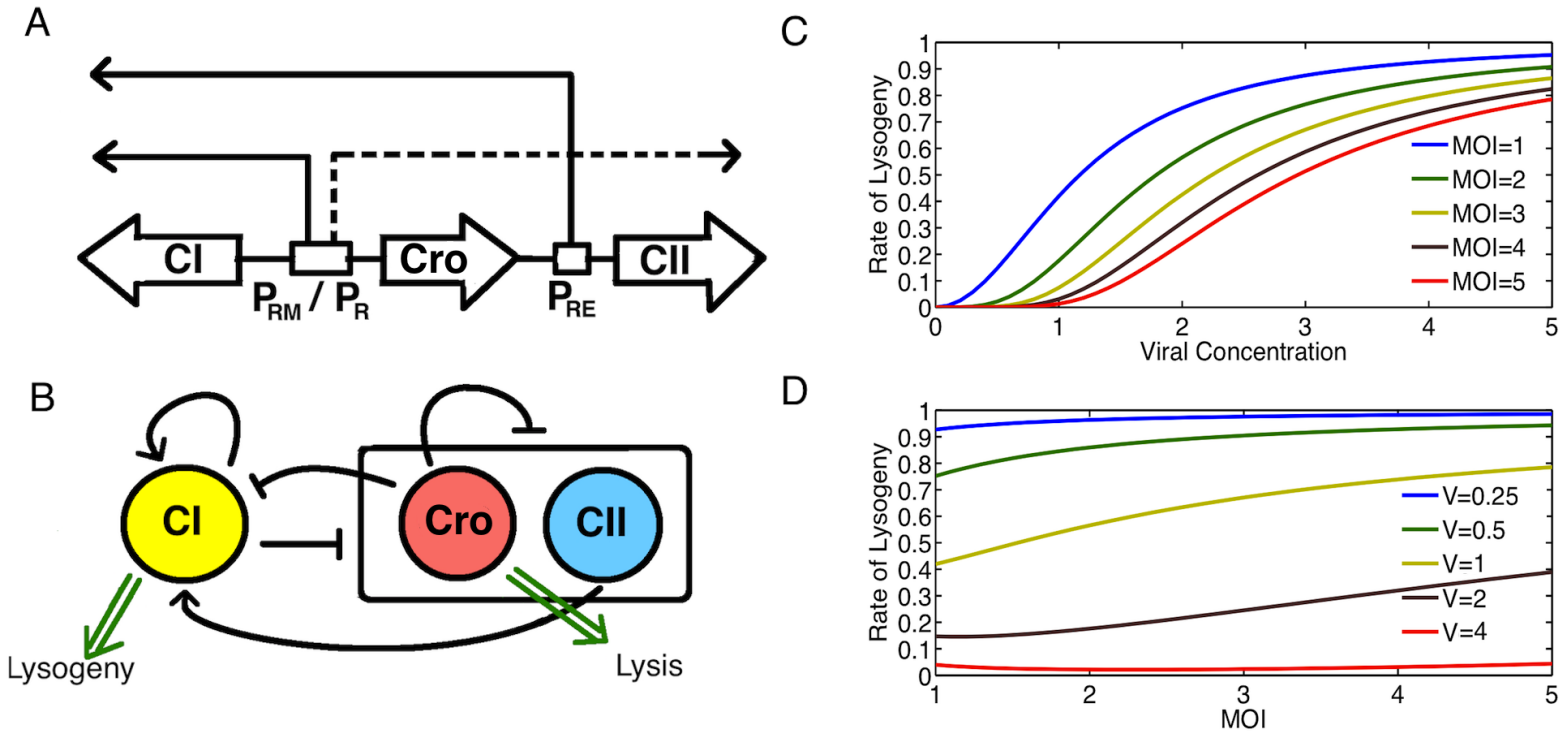
Decision Making in lambda phage. A: The role of the 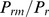 and 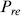 promoters involved in lambda phage decision. Dashed lines denote transcriptional events that require no activation while solid lines denote transcriptional events that require activation. B: Schematic of the core genetic network involved in lysis-lysogeny decision. CI gene promotes itself and represses the other genes. Cro represses everything, while CII promotes CI. C: Rate of lysogeny as observed from experimental observations in Zeng et al. [24] as a function of viral concentration for different MOI. D: Rate of lysogeny as a function of MOI for different volumes. Bacterial volumes and viral concentrations are expressed in arbitrary units using the normalized cell lengths following Zeng et al. [24].

To address the role of multiplicity of infection (MOI) in cellular decision making, we employ a relatively simple model of the lambda phage genetic circuit [12] (Fig. 1A and Methods). The gene regulatory network is composed of interlocked positive and negative feedback loops involving three early viral genes CI, Cro and CII (Figure 1B). There are multiple factors involved in the decision making process, but it is believed that the CII protein has an important role [8], [12]. High levels of CII promotes production of CI and lysogeny, whereas low levels of CII promotes production of Cro and bacterial lysis.

Zeng et al. [24] observe that the probability of lysogeny can be well described by the phenomenological function 
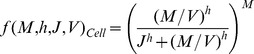
where 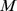 is the MOI, 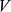 is the cell volume, 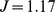 is the half saturation constant and 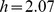 is the Hill number. In Figure 1C, we have plotted probability of lysogeny as a function of viral concentration (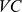), for different MOIs. It is evident that the probability of lysogeny at a given 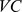 could be quite different depending on MOI; a small cell with MOI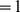 can have significantly larger probability of lysogeny than a cell with double the volume and MOI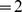, even though both have the same 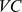. In the following, we will use the above mentioned case to investigate mechanisms that can explain the direct dependence of probability of lysogeny on MOI [24]. We note that the observation of weak dependence of the probability of lysogeny on MOI at fixed cell volumes as seen in Figure 1D can also be attributed to the existence of a partial dose-compensation mechanism [14]. We also note that the specific form of this phenomenological relation does not necessarily have mechanistic origin.

**Figure 2:**
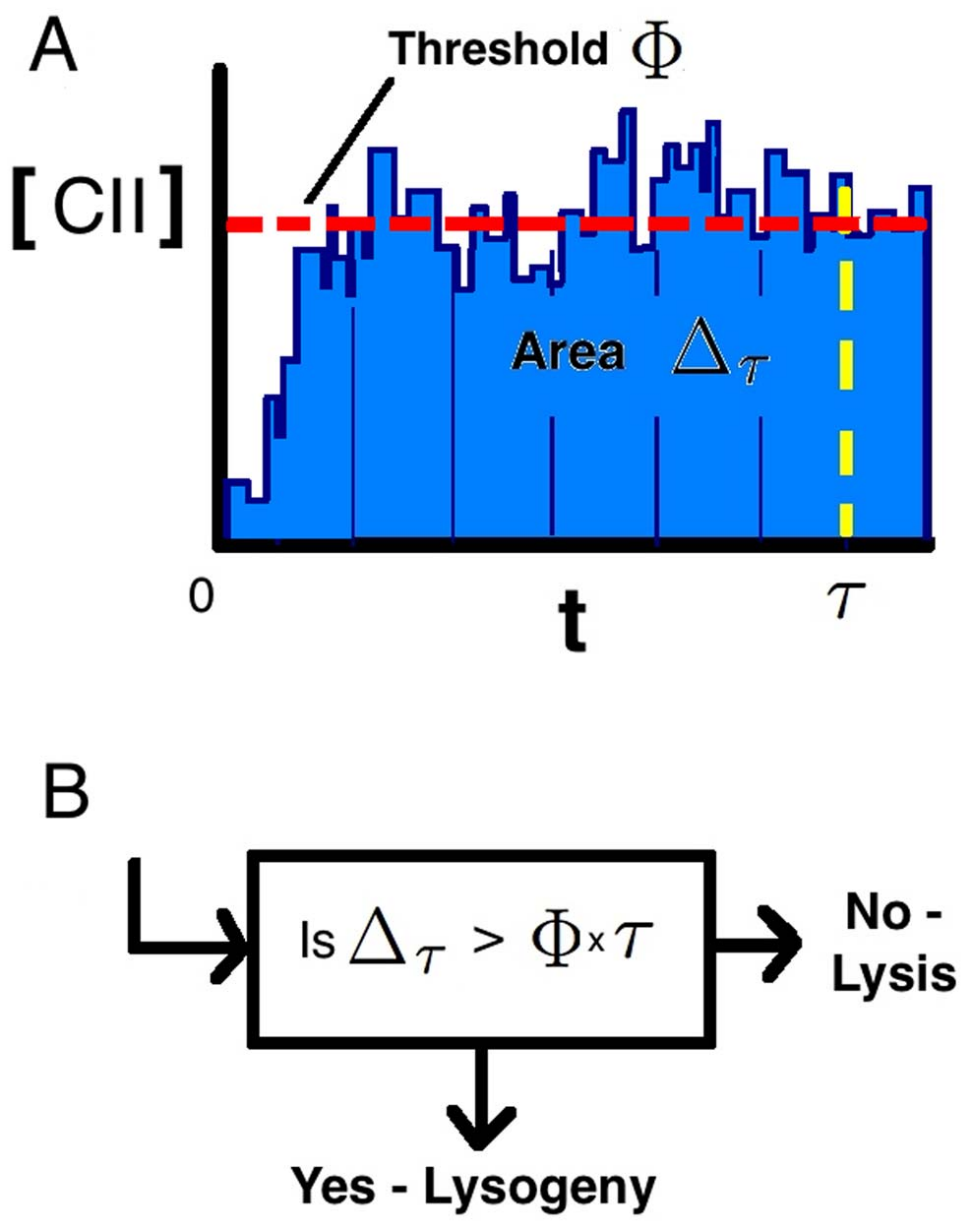
Decision Criteria. (A) Illustration of a stochastic trajectory of CII proteins from time of infection to decision. We consider the decision criteria as to whether the area under the curve of CII (light blue area) is above 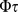, or equivalently average 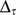 is above 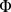 over a particular length of time 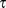. This criteria is outlined in flow diagram B.

To determine the outcome of infection *in silico*, we use stochastic simulations of our model to produce stochastic realisations of the early viral gene expression using the Gillespie algorithm [25]. To quantify the cell fate decision, we need to choose a criterion which we can use to classify whether an individual simulation undergoes lysis or lysogeny. The lambda phage decision is complex and could depend on transient or steady-state dynamics of multiple factors [11]. To unravel the basic mechanisms at work, we simply assume that the decision can be determined from the transient dynamics of a single gene, CII (Fig. 2A). As illustrated in Figure 2B, we assume if the time-averaged levels of CII up to a time point 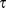 (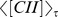) is below (above) a certain threshold 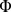 the cell fate is lysis (lysogeny). Observing CII levels at a single time point instead of time averaging to determine cell fate, produces very similar results (results not shown).

### Decision threshold and intrinsic noise

**Figure 3:**
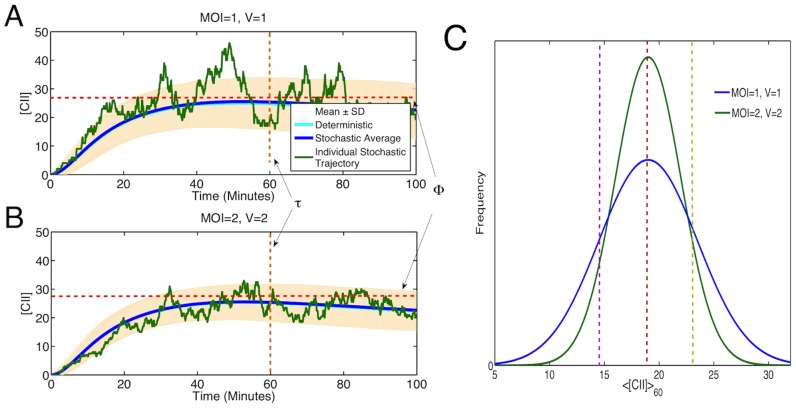
Average Stochastic Trajectories of [CII] over time interval 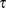 for stochastic non-spatial model. Mean trajectories shown (blue line) 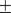 standard deviations (cream shaded region), alongside the deterministic trajectory (turquoise line). One stochastic trajectory from the data is also shown in green. (A) MOI = 1, V = 1 (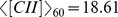,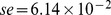). (B) MOI = 2, V = 2 (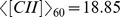, 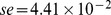). Results calculated based on 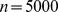 simulations. (C) Distribution of 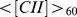 showing a low threshold (purple dashed line), threshold at mean (brown dashed line) and high threshold (yellow dashed line). This illustrates that the area under the curve exceeding the threshold is larger for MOI = 2, V = 2 for the low threshold, equal areas for the threshold at the mean and larger area for MOI = 1, V = 1 for the high threshold.

**Figure 4:**
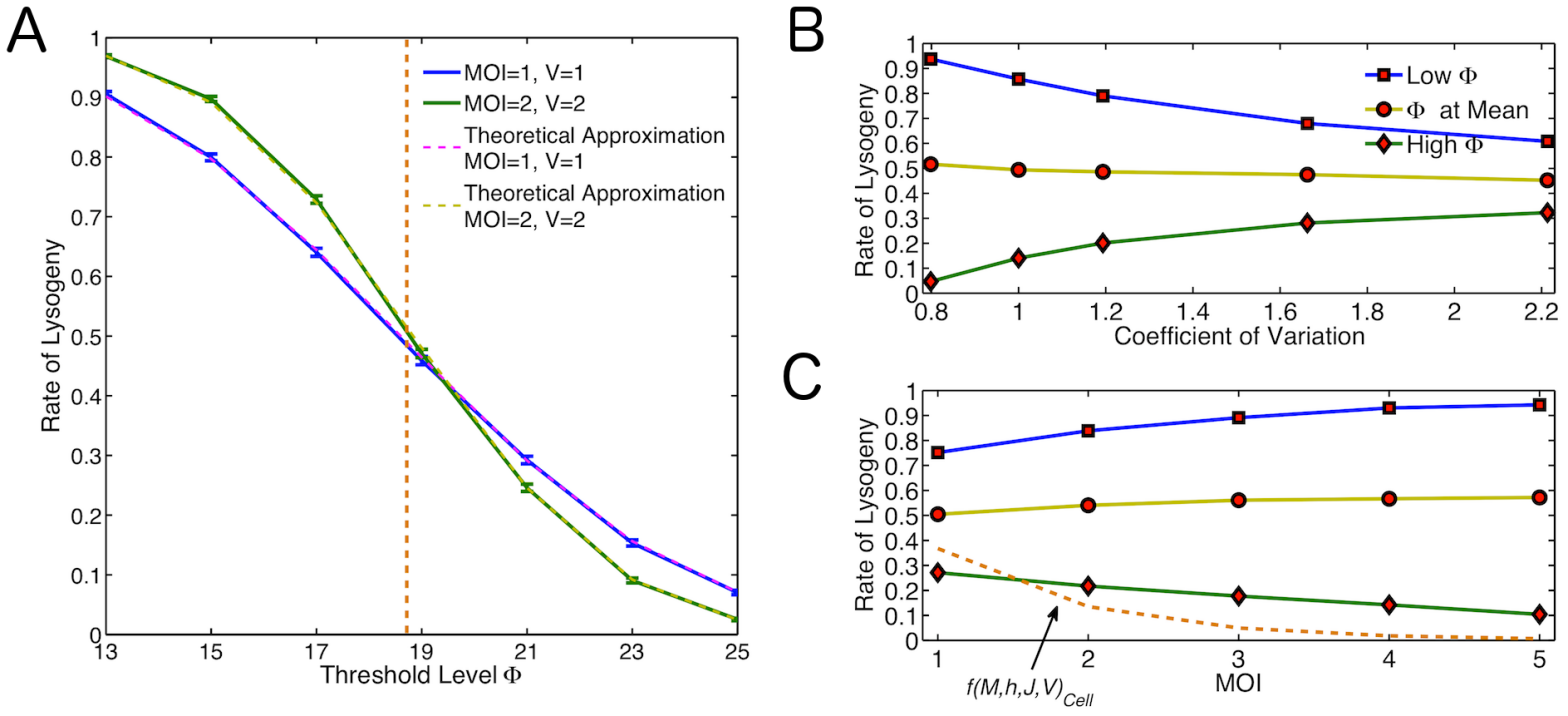
How the Relationship Between Noise and Threshold Level Affect the Decision. (A) Rate of lysogeny across different threshold values for basic model. Blue line: MOI = 1, V = 1; Green line: MOI = 2, V = 2; Orange dashed line: 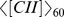; Pink and yellow dashed lines: Theoretical approximations for the rate for MOI = 1, V = 1 and MOI = 2, V = 2 respectively. (B) How changing the level of noise for same MOI affects the outcome at a high threshold (green line, 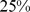 above the mean), threshold at 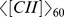 (yellow line) and low threshold (blue line, 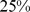 below the mean). (C) How the MOI affects the rate of lysogeny at a high threshold (green line, 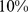 above the mean), threshold at 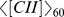 (yellow line) and low threshold (blue line, 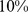 below the mean). The phenomenological rate 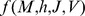 is shown for a typical unit cell volume using 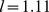. (orange dashed line).

In order to obtain insights to the role of MOI on the decision making process, we focus initially on the difference between cells with MOI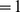 and MOI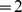 at VC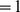 (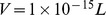). Figure 3 A and B show traces of a single stochastic trajectory and of the average [CII] based on 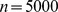 stochastic simulations. We find similar mean [CII] for both case of MOI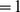, V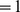 (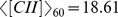, 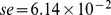) and MOI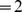, V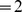 (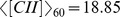, 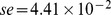). However, as shown in Figure 3 variation in the MOI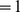 case is significantly larger than for MOI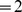, since it has more intrinsic noise due to having lower copy numbers of genes and other biomolecules. This is demonstrated further in Figure 3C. The rate of lysogeny is determined by the specific choice of the decision threshold 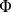 and time point 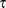. We choose 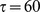 minutes, which is a reasonable choice given the timescales in which decisions occur as observed in [24]. However, our results are not sensitive to specific choice of 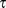 (results not shown). If we choose 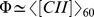 we find similar probabilities of lysogeny for the two cases. Interestingly, we find that for 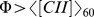 the rate of lysogeny is higher for MOI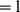 than for MOI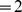, whereas for 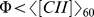 the rate of lysogeny is higher for MOI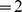 than for MOI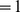 (Figure 4A). Since the average CII is similar for the MOI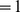 and MOI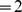 cases, the observed difference in the rate of lysogeny should be due to different levels of noise in CII. With larger noise a higher (lower) threshold than the average is exceeded more (less) frequently, as illustrated in Figure 3C.

To illustrate the role of molecular noise in the decision making process, we perform a simple analytical estimate based on a more simple decision criteria which uses a single time point observation. If we assume a Gaussian distribution for 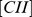 at time 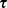 (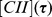) with mean 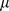 and standard deviation 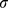, then



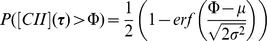
.

This inverse cumulative function is equivalent to the rate of lysogeny in this case. The error function (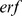) changes sign with that of its argument 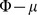. This accounts for the change in observed dependence on 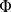 in relation to the mean CII. The size of this difference is then dictated by 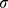, which is a measure of noise in CII. Therefore, it can be seen that an increase in MOI at constant 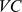, while it does not change 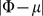, it decreases the denominator (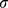) which affects the rate of lysogeny. The dashed line in Figure 4A shows the rate of lysogeny calculated from the above relation, which is very close to the result obtained in the simulations by using the 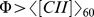 as a decision.

In order to further investigate the interplay between the decision threshold 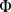 and the amount of noise in CII, independent of MOI and volume, we adjust the parameters to make the system more or less noisy. We therefore look only at the case where MOI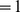, 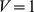, but specifically increase (decrease) noise by increasing (decreasing) translation and mRNA degradation rates, changing the burstiness of the gene expression [26] (Figure 4B). It is seen again that for higher levels of noise we see higher rates of lysogeny for 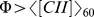 and lower rates of lysogeny for 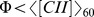 in accordance with the above reasoning. A similar trend is observed if we change the noise in CII by adding extrinsic noise via introduction of fluctuations in the kinetic rates [16] (results are not shown).

Based on the above argument, it follows that the trend in rate of lysogeny should be even stronger for higher MOI's at the same viral concentration. We therefore perform additional simulations for MOI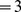,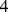,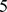 at 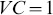. We find that the observed trend indeed holds for higher MOI's (Figure 4C). The reduction in noise at larger MOI is due to the increase in copy numbers for mRNA and proteins. The case of high threshold exhibits a trend similar to what is observed experimentally. Comparing these results with the phenomenological function 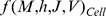 [24] suggests the decrease in gene expression noise at higher MOI could explain about half of the observed dependence of rate of lysogeny on MOI.

**Figure 5:**
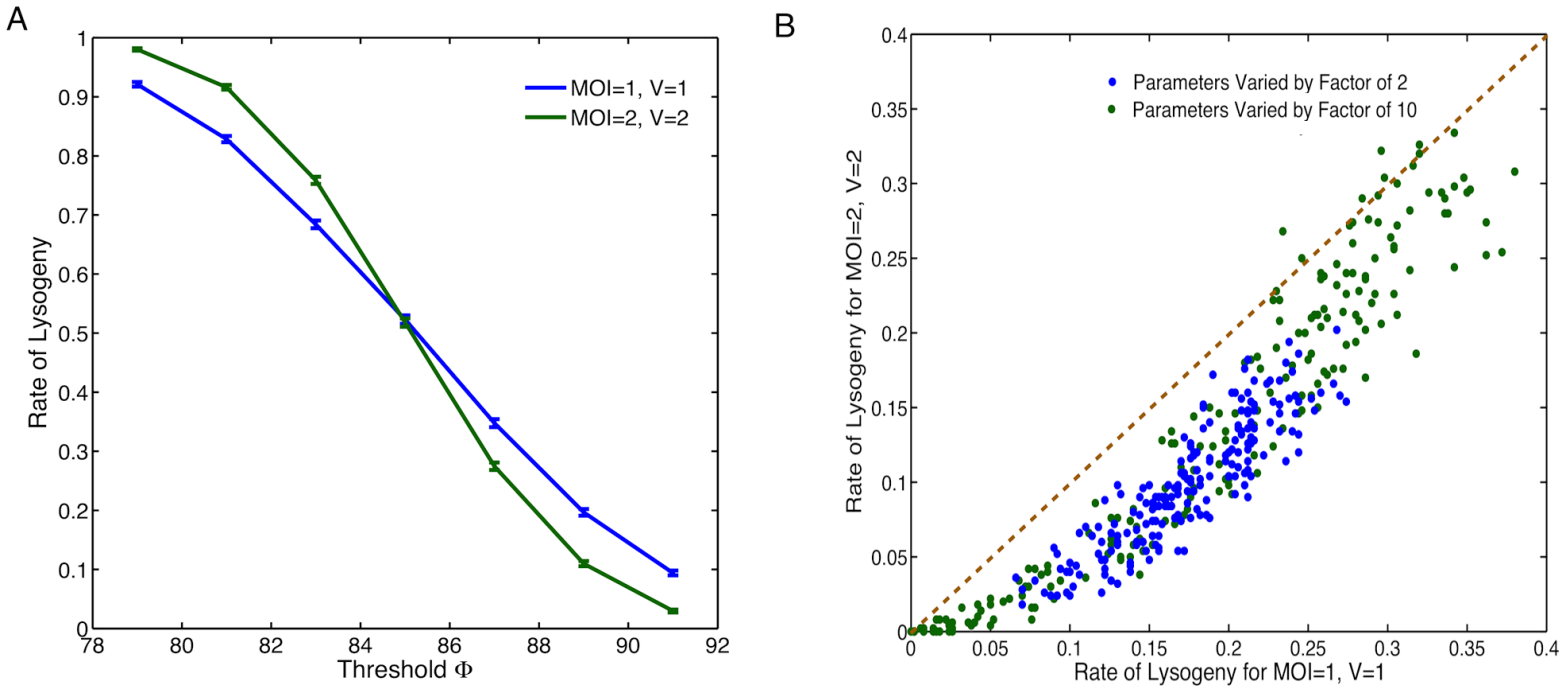
Robustness of our results with respect to variations in model assumptions and parameters. (A) Rate of lysogeny across different threshold values for model with CII tetramers instead of dimers, phage replication (deterministically doubling every 3 minutes for the first 15 minutes) and CI self repression. Blue line: MOI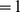, V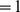; green line: MOI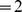, V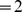. (B) Global sensitivity analysis using 200 different parameter sets chosen by randomly varying all parameters within a factor of 2 or 10 of their nominal values. Rate of lysogeny for MOI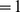, V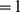 against MOI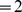, V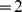 are plotted with blue dots for changes by a factor of 2 and green dots for changes by a factor of 10. The orange dashed line represents the point where rate for MOI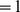, V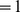 is equal to MOI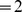, V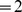. The rate of lysogeny for each parameter set is estimated using 500 stochastic simulations with a decision threshold set at 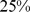 above the mean 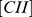 value for that parameter set.

The proposed role of intrinsic noise in decision making is general and does not depend on the specific choice of model assumptions and parameters. To explicitly demonstrate the general validity of our results, we have performed additional analysis including parameter sensitivity analysis and modifying some of our modelling assumptions. Firstly, our model assumes CII is a dimer for simplicity, while it is known that CII is in fact tetrameric [27]. We therefore look at the effect of allowing CII dimers to bind and form tetramers, and tetramers to bind to the promoter and controlling gene expression. Secondly, there is evidence that upon infection phages replicate in the cells doubling their number every 2–3 minutes for the first 15 minutes [28]. Including phage replication in our model has the effect of increasing the mean [CII]. While CI can undergo self repression at higher concentrations of the dimer [11]. We attempted to include all of these assumptions in the model, and the results are shown in Figure 5A. It can be seen that adding these features, despite a change in the mean [CII], we observe similar qualitative results. We also tried including each of modifications individually, observing again that our conclusions still hold (results not shown). Finally, to test the effect of model parameters, we performed a global parameter sensitivity analysis on our system by varying all model parameters randomly within a factor of two or ten below and above their nominal values. For almost all parameter sets tested, using a high decision threshold (greater than mean 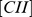 for that parameter set), we observed a lower rate of lysogeny for the case MOI = 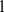, 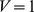 compared to the case MOI = 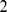, 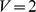 (Figure 5B), which is consistent with what is observed for the original parameter set (Figure 4C).

### Spatial Effects

**Figure 6:**
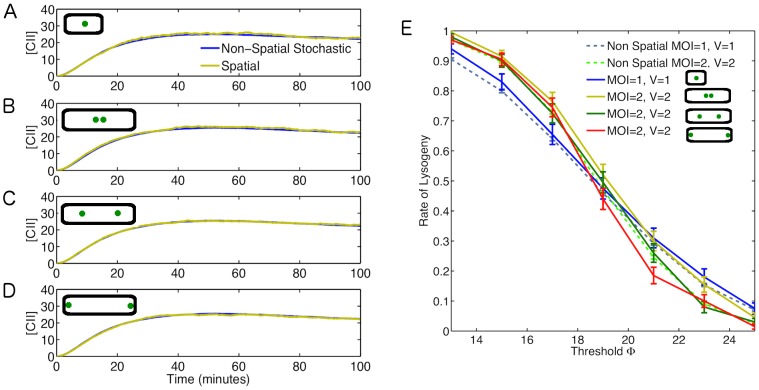
The Role of Spatial Effects. Average CII concentrations for different rates of diffusion. (A) MOI = 1, V = 1, phage at centre (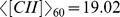,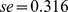); (B) MOI = 2, V = 2, phage at centre (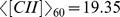,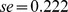); (C) MOI = 2, V = 2, phages at a quarter and three quarter of the cell length (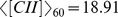,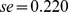); (D) MOI = 2, V = 2, phage at cell poles (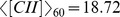,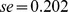). Average [CII] with for non-spatial model (blue line) shown alongside spatial model (yellow lines). Results based on 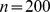 simulations. (E) Rate of lysogeny across different threshold values for spatial model with original diffusion rates (solid lines). Results are shown alongside the non-spatial results (dashed lines).

Phage can infect bacteria at any position along the cell surface, although recent research suggests that phage prefers the poles [24], [29]. Thus, it is likely that multiple phage are spatially separated in the bacteria. Delay caused by the diffusion of biomolecules from one phage to another could affect the rate of lysogeny, particularly when diffusion is slow or the infecting phages are far apart. To investigate possible spatial effects on rate of lysogeny, we use a particle-based approach that tracks individual molecules as they diffuse and react inside the cell [30]. We compare the case where there is one infecting phage positioned at the centre of a small cell with the case where two phage arranged in 3 different ways infect a cell with double the volume, therefore keeping viral concentration the same. In these cases two phages will be positioned either at the centre, at a quarter and three quarter of the cell length or at the cell poles (Figure 6A–D). The results were also compared with the non-spatial stochastic simulations outlined in the previous section. Due to the computational time required to do these simulations we perform a lower number in comparison to the non-spatial case (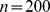), still the standard error is relatively low. It can be seen in Figure 6A–D that the effect of phage positioning and diffusion rate on the mean [CII] is small. These results are also close to the non-spatial and deterministic models.

It can be seen in Figure 6E that there are some effects on rate of lysogeny due to phage positioning. Specifically the rate is lower (higher) for the case when phages are at the cell poles for 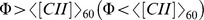. While the rate is highest (lowest) for cells with phages at the centre when 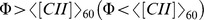. The reason for this could be the small difference in mean [CII] observed. The case where phages are equally spaced is in general an intermediate of the other results and follows the non-spatial results the closest. In comparison with the non-spatial results the effects of phage positioning are not as important as intrinsic noise. In most cases the spatial results are not significantly different from their non-spatial counterparts. It should also be noted that if we were to average the 3 cases when MOI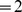, then the rates are very close to the non-spatial case. Therefore, this is only likely to be of any noticeable effect if there were any bias in the positioning of the phage. While experiments [24], [31] have displayed some infection site bias to the cell pole, it it unlikely to be enough to solely explain the observed difference in rate of lysogeny.

### Cell Growth Effects

**Figure 7:**
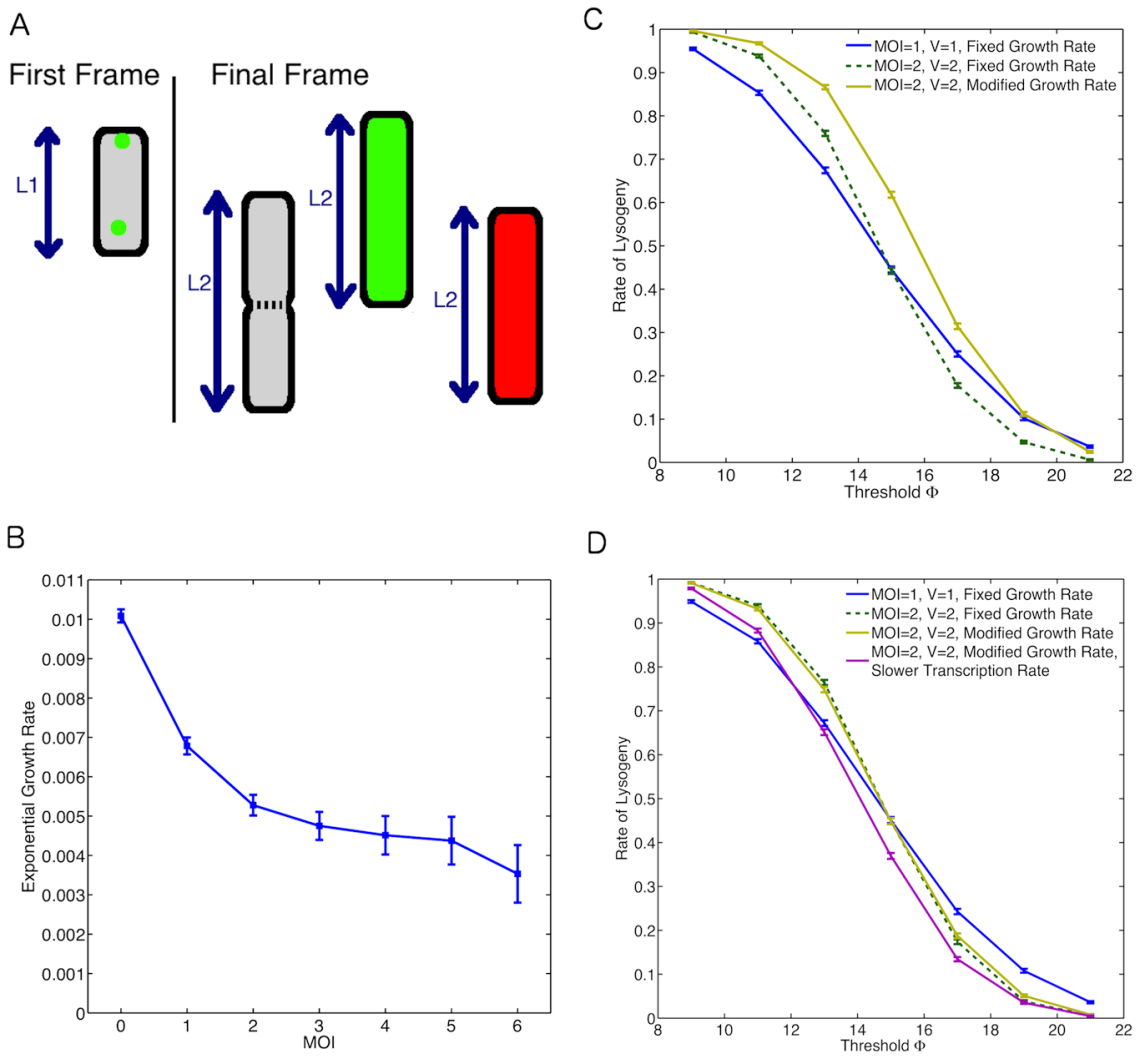
The Role of Cell Cycle Effects. (A) Method for determining cell growth rate. Measurements are taken at 2 time points, the first frame and final frame. The final frame is determined by the event. For an uninfected cell (grey) this is the point at which the cell divides. For an infected cell it is the point at which a decision of lysis (green) or lysogeny (red) has been made. The growth rate was calculated using 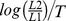 where 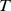 is the time between the first frame and the final frame. (B) Observed growth rates at different MOI. (C) Effect of cell growth on rate of lysogeny by considering dilution only. Fast rate 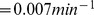 and slow rate 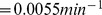. (D) Effect of growth rate on rate of lysogeny when also considering possible changes in transcription rates. Growth rate modulation of transcription rate are similar to the study by Klumpp *et al.* [33], with a two fold enhancement in the slower transcription rate case.

MOI could affect the decision making process by influencing the general physiology of the bacteria. To investigate this issue, we reanalysed the original movie data from [24] (courtesy of Lanying Zeng and Ido Golding). Specifically, we looked at the effect of MOI on growth rate (elongation rate) of the cell. Figure 7A illustrates how we have estimated the cell cycle time. Cells were observed from time of infection until a decision event or first cell division. It is observed that cells with higher MOI have lower growth rate (Figure 7B). In [24], it was found that cells with higher MOI had a larger proportion of non-growing cells, which is consistent with our results. We note that in estimating the growth rate of cells we have removed all non-growing cells from our analysis.

This information on growth dependency at different MOI is then incorporated into our model. We therefore accordingly adjust the dilution rates [32] to mimic a cell growing at different rates. We compare the results with those of the non-growing cells and also growing cells with no effect of MOI on growth rate. It can be seen in Figure 7C that accounting for the growth effects observed experimentally by only modulating dilution rate actually goes against the dependence on MOI observed experimentally. This is likely due to the increased relative volume in accordance with the dependence of rate of lysogeny on volume [23].

It is known that cell growth rate controls other gene expression parameters in addition to dilution rate [33] for example by regulating the rate of mRNA transcription [34], [35]. We therefore looked at the effect of coupling the rates of mRNA transcription to the growth rates on the decision making process. It can be seen that this increases the average protein concentration and therefore affects the rate of lysogeny (Figure 7D). Specifically, since the growth rate for cells with MOI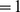 is higher than those with MOI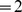, then 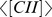 would be larger in the case where MOI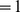 due to the increased mRNA transcription. Thus this increases the rate of lysogeny and the difference in rates concurs with the experimental results on MOI dependence of rate of lysogeny. Therefore, if the transcription rate increases sufficiently at higher growth rates then cell growth modulation by MOI could also be a contributing factor.

**Figure 8:**
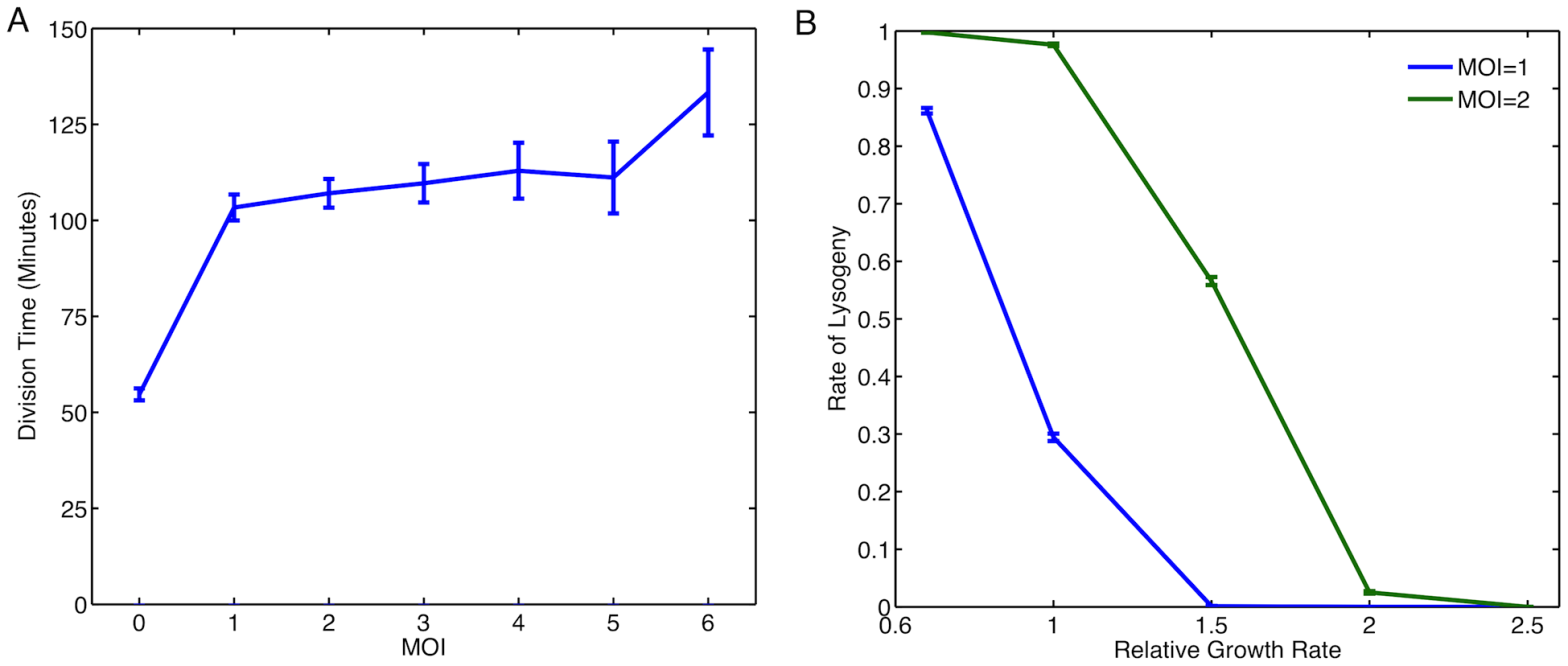
The Effect of Growth Media on Rate of Lysogeny. (A) Division time for cells infected with lambda phage. For MOI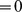 this is the division time of the cell, for MOI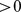 this is the division time for infected cells that choose lysogeny. (B) Effect of growth rate on rate of lysogeny in different growth media. Here, simulations compared the rate of lysogeny in cells of the same size with different MOI. Growth rate modulation of transcription rate are similar to the study by Klumpp *et al.* [33].

Growth media can modulate bacterial growth rate (elongation rate) and cell cycle time [33]. Since, we observed MOI modulation of growth rate affects rates of lysogeny, we argued growth modulation by growth media should have similar effects. To investigate this phenomenon using our model, we assume modulation of cellular growth rate does not reduce cell cycle time below 60 minutes over which we estimate the rate of lysogeny. This is a reasonable assumption since in the analysis of experimental movies from [24], we observe that phage infection delays division time (in the generation of cells that are infected) for cells that undergo lysogeny (Figure 8A). We use growth rate modulation of transcription rate and dilution to estimate rate of lysogeny for the MOI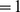 and MOI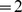 in a cell of the same size (Figure 8B). Note that the rate of lysogeny is higher for MOI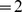 due to the higher viral concentration. The model predicts that a decrease in growth rate will lead to sharp increase in rate of lysogeny. We also observe that apart from the extreme points, where the rate of lysogeny is close to 0 or 1, that the observed difference between MOI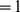 and MOI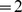 is largely preserved (Figure 8B).

## Discussion

Recent experimental results suggest the rate of lysogeny in lambda phage infection depends not only on 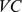 but also on the MOI directly [24]. Using a simple model of genetic networks controlling this fate decision, we have demonstrated that intrinsic noise is a determining factor of the rate of lysogeny in lambda phage. Viral gene dynamics are more noisy for small MOI due to low copy number of viral promoters. A viral protein with higher fluctuating dynamics reaches a high decision threshold more frequently, producing a direct dependence to MOI and its associated intrinsic noise. Although we observed some effect due to spatial segregation of the multiple infecting phage, this is not sufficient to solely explain the observed dependence on MOI. New analysis of the existing experimental movies revealed cell growth dependence on MOI potentially affecting the decision making process. Including the growth rate dependence and its effect on transcription rates in our model suggest another possible contribution to the observed MOI dependence. We conclude that a combination of the proposed mechanisms contribute to the explanation of the observed dependence of decision rate on MOI in the lambda phage system.

The model we use is simple, but captures the essential features of the lambda phage system [13]. The model does not accurately reflect the CII levels on the time scale considered here. Experimental observations suggest that CII levels fall dramatically by 60 minutes after infection but they remain high in our simulations due to the simplicity of the model and parameters used. Also, our choice of a single decision threshold is crude. However, our results agree qualitatively with work that use more complex criteria [14]. Our main result on the role of intrinsic noise in the decision making process is quite general and is not dependent on the specific choice of the genetic model assumptions, model parameters or decision criteria. We show that modifying model parameters or some of the model assumptions does not affect our conclusions. In general, any non-linear decision making process should depend on both the average dynamics of the viral genes and their fluctuations. While the average behaviour is mainly controlled by the viral concentration (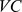), the fluctuations are dependent on the absolute copy number of biomolecules and therefore the number of infecting phage (*i.e.* MOI). The role of gene expression noise on the cell fate decision making process has been investigated in other systems [36]–[41]. Our results add to the growing body of work that suggest stochastic effects have significant influence in the decision making process [17], [18].

We used a particle-based spatial Monte Carlo approach to investigate the effect of spatial segregation and diffusion on the decision making process. We find that spatial segregation introduces small but non-zero effects on the rate of lysogeny. This is because the mean level of viral protein CII is dependent on the spatial arrangement of the phage particles inside the cell. Based on the physiological diffusion coefficients we have used (Table 1), viral mRNAs travel across a typical sized cell in about 10 minutes and it takes only few seconds for viral proteins, while the time-scale of decision making is much longer set at 60 minutes in this study. Of course, presence of diffusion barriers can significantly increase the effect of spatial segregation on the phages inside the cell. We note that diffusion could have subtle effects on gene expression noise [42] and this in turn could also affect the decision probabilities as discussed above.

**Table 1:** Model Parameters.

Parameter	Description	Value
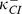	Backward dimerisation rate of CI	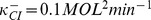
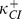	Dimerisation rate of CI	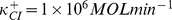
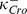	Backward dimerisation rate of Cro	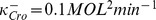
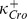	Dimerisation rate of Cro	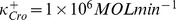
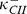	Backward dimerisation rate of CII	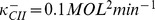
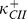	Dimerisation rate of CII	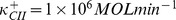
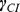	Decay Rate of CI	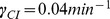
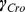	Decay Rate of Cro	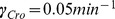
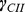	Decay Rate of CII	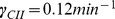
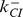	Protein Unbinding Rate of CI	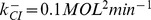
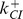	Protein Binding Rate of CI	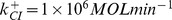
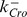	Protein Unbinding Rate of Cro	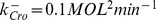
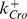	Protein Binding Rate of Cro	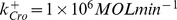
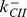	Protein Unbinding Rate of CII	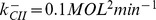
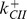	Protein Binding Rate of CII	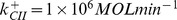
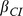	Transcription rate of mRNA for CI from promoter 1	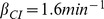
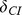	Transcription rate of mRNA for CI from promoter 2	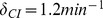
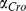	Transcription rate of mRNA for Cro	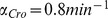
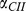	Transcription rate of mRNA for CII	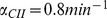
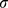	Translation rate	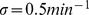
	Time step (Spatial Simulations)	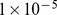
	Protein Diffusion Coefficient	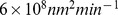
	mRNA Diffusion Coefficient	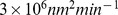
	Binding Radius (Spatial Simulations)	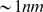
	Cell Width (Spatial Simulations)	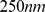

Image analysis of the original movies from the study by Zeng et al. [24] reveals that the cell growth rate decreases at higher MOI. This is probably due to the additional strain on the cellular resources needed to support multiple phage. It would be interesting to investigate if there are similar effects of MOI on the growth rate of lysogens and possible repercussions for the stability of the genetic switch. We observe that including the MOI dependence of cellular growth rate combined with growth rate regulation of transcription in our model can produce a contribution to MOI dependence of rate of lysogeny.

In addition to what is described above, we have investigated other potential influencing factors that could provide explanations for the observed results. While experimentally, infections were synchronised by a method of temperature change (described in [24]), we analysed the effect of a small difference in infection times for multiple phages. When comparing a cell of MOI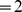 with phage infecting at the same time against a cell with a small difference in the timing of the infecting phage, we observed a small difference in mean 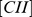 but no significant difference in the proportion choosing lysogeny. Another possible factor could be the crude estimation of the cell volume based on cell length since due to curved ending of *E. coli*, double the length will not be exactly double the volume. Performing simulations comparing cells that accounted for this small offset in volume, only slight differences in mean 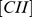 were observed and it was clear that noise was still the dominating factor on the outcome.

To experimentally test if the proposed mechanisms in this study can explain the MOI dependence of rate of lysogeny as observed by Zeng et al. [24], we propose the following experiments. Our model predicts that larger viral gene expression noise increases rate of lysogeny (Figure 4B). To test the role of noise in lambda phage decision making, firstly one needs to measure cell-to-cell variability in viral gene expression noise. In addition, re-engineering lamda phage for example by modifying promoter strength or mRNA stability to modify gene expression noise can validate the role of intrinsic noise in lysis-lysogeny decision making. To test the role of cellular growth on phage decisions, quantifying rate of lysogeny in infected bacteria growing in different growth media can validate our prediction of strong dependence of decision outcome to cellular growth as shown in Figure 8B. Together, these experiments can elucidate the proportional contribution of the proposed mechanisms underlying the observed MOI dependence of phage decision making.

Partial gene dosage compensation is a widely observed phenomena in cellular systems, however a complete understanding of the underlying mechanisms is lacking [43]–[46]. Previous studies have suggested that volume changes [43], nonlinear biochemical interactions [46] and network structure [45] may contribute to this compensating effect. As proposed by Joh and Weitz [14], gene dosage compensation can provide an explanation for the observed MOI dependence of rate of lysogeny. Thus, the factors that we have investigated influencing the phage decision also provide potential general mechanisms for gene dosage compensation. Intrinsic gene expression noise is dependent on gene dosage and therefore, if coupled to nonlinear dynamics has the potential to play a part in all forms of gene dosage effects. Spatial segregation of genes and diffusion can also contribute to a gene dosage dependent phenomenon particularly in larger cells. Finally, any effect of gene dosage on cellular growth rate can be the basis of gene dosage dependent phenomenon [43]. It is likely that a combination of these factors can contribute towards partial gene dosage compensation in biological systems.

It is thought that the lysogenic state is a form of defence mechanism to prevent extinction [4]. It is not clear why the form of MOI dependence observed in [24] is beneficial for the phage in this context. Our exploration on this issue thus far has revealed ecological dynamics under fixed resource conditions does not show clear benefit for this specific form of decision over a constant rate of lysogeny that does not depend on the physiological state of the cell. It is possible that the advantage of this form of decision making is only seen in fluctuating environments [47], and this could be an interesting avenue for future research.

## Methods

### Model

The model that is used in this study focuses on the dynamics of early viral genes. This representation of the system originally appeared in [12]. It includes the CI, CII and Cro genes which are important in determining the outcome of the lysis-lysogeny decision. These are generated by the 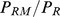 and 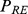 promoters. The equations are shown below
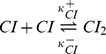


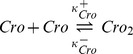


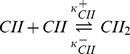


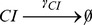


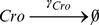


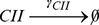


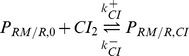


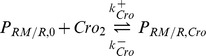


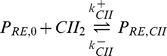


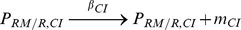


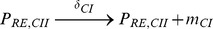


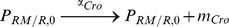


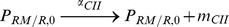


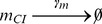


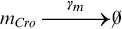


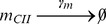


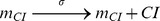


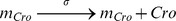


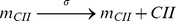
where 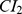, 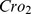 and 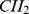 represent the dimers of 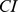, 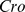 and 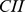 respectively, while 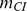, 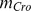 and 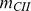 represent their corresponding mRNA. The unbound promoters are represented by 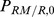 and 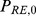, and 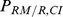, 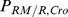 and 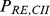 denote the bound configurations of the promoters. The parameters used are shown in Table 1 and are the same as originally used in [12]. Dimerisation rates are given by 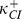, 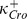 and 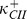 while the corresponding separation rates are given by 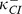, 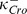 and 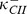. Transcription rate of 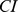 from 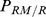 is given by 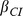 and from 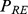 it is given by 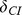. Transcription rates of 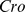 and 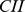 are given by 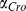 and 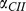 respectively. The decay rates of the proteins are given by 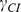, 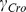 and 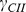 respectively. Translation rates of all proteins are given by 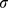. The binding rates of dimers to promoters are given by 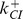, 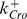 and 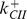 while their unbinding rates are given by 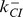, 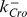 and 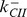. In contrast to the original deterministic analysis of this model [12], we simulate this model stochastically using Facile [48] and Easystoch [49]. The approach used the Gibson-Bruck [50] implementation of the Gillespie algorithm. Spatial modelling was carried using a Monte Carlo particle-based tool, Smoldyn [30]. The diffusion constants used were found using Bionumbers [51] and are shown in Table 1. For both spatial and non-spatial simulations, data points were output at every 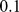 minutes. The analysis of the generated data was carried out in Matlab (version R2012a). The means protein numbers were calculated over all output times between 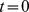 and 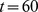 minutes. The concentrations were calculated by dividing the protein numbers by the standardised cell volumes where V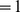 corresponds with a volume of 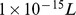. The mean concentration of each run of the system was then compared to a threshold level 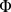 to determine whether the outcome was lysis or lysogeny.

### Cell Image Analysis

Time lapse cell imaging analysis, and subsequent statistical analysis was performed in Schintzcell (courtesy of Michael Elowitz, [52]) and Matlab respectively. For details of the time lapse microscopy and other experimental details, see Zeng et al. [24]. Cell length was measured in the first frame and when they were deemed to have made a lysis or lysogeny decision. This decision point was determined by the a threshold level or YFP (lysis) or mCherry (lysogeny) which, once crossed meant the cells were committed to the chosen outcome. The number of infecting phage were counted in the first frame stack using ImageJ (http://rsbweb.nih.gov/ij/).
